# Tongue base varix as a source of oral bleeding: A case report

**DOI:** 10.1097/MD.0000000000016987

**Published:** 2019-10-18

**Authors:** Young-Mo Kim, Ji Won Kim, In Suh Park, Jeong-Seok Choi

**Affiliations:** a Department of Otorhinolaryngology-Head and Neck Surgery; b Department of Pathology, Inha University School of Medicine, Incheon, Republic of Korea.

**Keywords:** oral bleeding, tongue base, varix

## Abstract

**Rationale:**

Oral bleeding is usually diagnosed after by referral to other department for the differential diagnosis of hematemesis or hemoptysis. If a patient presents with blood in the oral cavity with no obvious source, generally upper airway, pulmonary, or gastroesophageal lesions are considered likely bleeding foci. The tongue base is an unusual site for laryngopharyngeal varices and only a few cases have been reported.

**Patient concerns:**

Although varix at the tongue base in patients with liver cirrhosis has been rarely described, physicians must consider variceal bleeding from the tongue base when presented with oral bleeding. In such cases, bleeding foci can be identified and controlled by laryngoscopy. We describe the case of a 42-year-old woman complaining of small amount of hemoptysis with variceal bleeding at the tongue base controlled by laryngoscopic excision and cauterization.

**Diagnosis:**

A diagnosis of tongue base varix was made based on medical history, clinical manifestations, laryngoscopic findings and pathologic features for the patient.

**Interventions:**

The successful laryngoscopic procedures were performed.

**Outcomes:**

The patient has shown no recurrent oral bleeding during follow-up.

**Lessons:**

Variceal bleeding in the tongue base is likely to cause serious massive hemorrhage. We need to consider this possibility when presented with a patient with intraoral bleeding but no evidence of hemoptysis or hematemesis.

## 1 Introduction

Oral bleeding is a common reason for visiting a general clinic, but bleeding from the tongue is rarely diagnosed in this setting. In many cases, such patients visit another department because oral bleeding is mistaken for hemoptysis or hematemesis. Sometimes, it is difficult to determine the location or cause of bleeding because the mouth and pharynx are covered with hemorrhage and hematoma during physical examinations.^[1]^ When a bleeding focus is confirmed, laryngoscopic procedures are used for diagnosis and hemostasis. Furthermore, in patients with liver cirrhosis, varicose veins are commonly found in the gastrointestinal tract, such as, in the esophagus or stomach, but are rare at the tongue base.^[2–4]^

We report the case of a woman without a history of liver cirrhosis who presented with oral bleeding of unknown origin. Esophagogastroduodenoscopy and chest computerized tomography (CT) showed no evidence of bleeding, but an additional oral examination located the bleeding focus at the tongue base. Bleeding control was achieved using laryngoscopic procedures. Patients have provided informed consent for publication of the case.

## 2 Case reports

A 42-year-old woman presented at our emergency department (ED) due to a small amount of hemoptysis of three days duration. She had habitually drunk alcohol on a daily basis for several years and her last medical checkup had revealed hypertension and liver enzyme elevation, but she was not evaluated further. She was not taking any anticoagulant medicine. Her vital signs on arrival included a temperature of 37.3°C, pulse 135 beats per minute, blood pressure 135/79 mmHg, respiratory rate 20 breaths per minute, and oxygen saturation 97% in room air. Laboratory results included hemoglobin 13.2 g/dL, platelets 99 K/mm^3^, international normalized ratio (INR) 1.13, and prothrombin time (PT) 14.5 seconds.

Physical examination revealed spider angioma in both the buccal area and neck, but digital rectal exam (DRE) and nasogastric tube irrigation were negative for gastrointestinal bleeding. Abdominal computed tomography was performed in the ED and revealed liver cirrhosis, fatty liver, and hepatosplenomegaly. ED doctors assumed the bleeding stemmed from Mallory-Weiss syndrome or an esophageal varix, and as a result, she was admitted to our gastrointestinal (GI) department.

Two days later, esophagogastroduodenoscopy (EGD) failed to detect any bleeding focus or varix lesion, and because symptoms seemed to have resolved the patient was discharged on close observation.

A week after discharge, she represented at our ED with hemoptysis. Nasogastric tube irrigation showed signs of active bleeding but this cleared after one-liter of saline irrigation. DRE results were negative. The patient was re-admitted and underwent follow-up EGD, but no bleeding focus or varix lesion was found in the esophagus, stomach, or duodenum.

**Figure 1:**
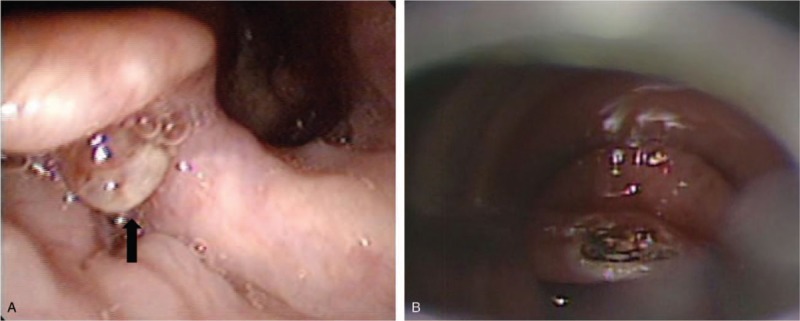
Endoscopic findings. Varix with an umbilicated surface in the tongue base (arrow) was exposed (A) and excised (B) using a laryngoscope.

**Figure 2:**
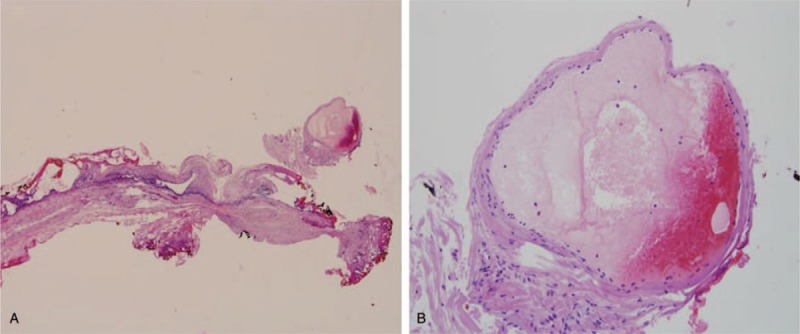
Histology of excised varix. The affected vessel was thin-walled, distended, filled with blood, and lined with squamous epithelium. (A: ×40, B: ×200, Hematoxylin and Eosin stained).

A pulmonology evaluation, which included chest computed tomography, produced no remarkable findings. Otorhinolaryngology was consulted and subsequent fiberoptic nasopharyngoscopy revealed a 0.5 cm sized umbilicated red “vessel like lesion” at the tongue base (Fig. 1A). Accordingly, we decided on emergency laryngoscopic microsurgery (LMS) under general anesthesia. In operation field, the lesion was completely removed using a CO_2_ laser (continuous mode, 2W) and sent to pathology (Fig. 1B). The periphery of the lesion was cauterized to prevent bleeding, and the operation was completed after confirming no further bleeding. The pathology report showed excised tissue was varix with acute thrombus lined with simple squamous epithelium with erythrocytes (Fig. 2).

**Figure 3:**
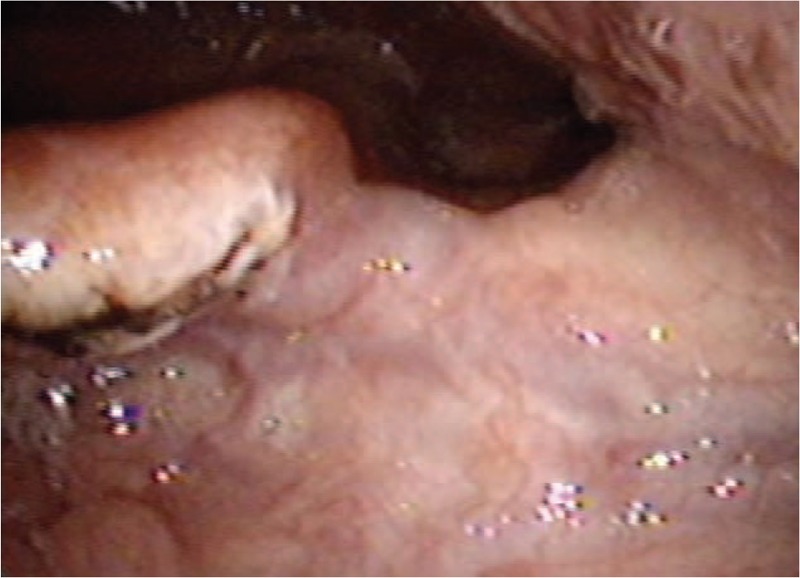
On post-operative day 2, laryngoscopy confirmed no bleeding or oozing at the location of the bleeding variceal lesion.

On post-operative day 2, follow-up fiberoptic nasopharyngoscopy confirmed no bleeding or oozing from the prior location of the variceal lesion (Fig. 3), and on post-operative day 3 the patient was discharged without complication. During 6-months of follow-up, she remained well with no evidence of recurrence.

## 3 Discussion

When a patient presents with intraoral bleeding but without a clear bleeding focus, it is important to determine whether the bleeding originates from the upper respiratory tract, gastrointestinal tract, or respiratory system, and thus, detailed history taking is critical.

The causes of hematemesis are peptic ulcer (35–50%), varicose veins (5–12%), esophagitis (20–30%), gastritis or duodenitis (10–20%), Mallory-Weiss laceration (2–5%), and tumors (2–5%).^[5]^ Hematemesis may be accompanied by nausea, a history of gastrointestinal disease, and/or dark coffee colored vomit, whereas in cases of hemoptysis, patients usually have a history of coughing and pulmonary disease, and produce hemorrhagic, foamy sputum.^[6]^ Oral bleeding is observed as relatively reddish hemorrhage, and the bleeding focus is more easily found after gargling. Sometimes, identifying the bleeding focus is made difficult by the presence of blood clots or the gag reflex. Nevertheless, in the pharyngeal area, a laryngoscope is useful for finding bleeding foci and for determining causes of bleeding.

Varix of the tongue base is extremely rare. The first case of varicose vein formation in the tongue base (vallecular fossa) region was first reported by Wetherill et al in 1967^[7]^ and more recently, Booton et al in 2002^[3]^ issued case reports on the topic.

Variceal bleeding in the tongue base is likely to be overlooked because bleeding is rarely encountered in this location, lesions are small, and hemorrhage is intermittent. However, variceal hemorrhage can sometimes cause serious massive hemorrhage. For this reason, we always consider this possibility at the tongue base when presented with a patient with intraoral bleeding but no history of lung disease or gastrointestinal disease and no evidence of hemoptysis or hematemesis.^[5,8]^

If a bleeding lesion is observed in the tongue base, complete excision and cauterization could be planned to achieve bleeding control. In the described case, we found laryngoscopic intervention both economical and useful for removing the lesion. On the other hand, if a lesion is located at the lateral aspect of the epiglottis with widely distributed vessels, a CO_2_ LASER has been reported to be effective for resection and to provide excellent access.^[9]^

Rapid, detailed history taking is important for predicting the location of bleeding lesions in patients with intraoral bleeding. Radiologic techniques, such as, computed tomography, laryngoscopy, and endoscopy may be necessary to determine etiologies, and in cases of massive hemorrhage, angiography is sometimes necessary. EGD usually involves entry through the esophagus at the posterior arytenoid cartilage. However, the tongue base is located in the lower part of the epiglottis and when pharyngeal hemorrhage occurs, blood often accumulates in this area, and as a result, many endoscopists overlook lesions of tongue base because endoscopes enter the esophagus directly and varicose veins and bleeding lesions may be missed. Accordingly, we advise when endoscopists perform an endoscopic examination of patients with intraoral bleeding, the circumference of the endoscopic entry site be carefully examined.

In addition, when planning bleeding control using laryngoscopy, surgeons should consider lesion location and size, the possibility of re-bleeding, how to access the lesion easily, and how to control the lesion, such as, by electrocauterization or by using a laser. In addition, a histologic examination is needed to differentiate these lesions from other masses, such as, cysts and vascular and malignant tumors.

In patients with oral bleeding with newly diagnosed cirrhosis by radiologic examination, additional examinations in addition to laryngoscopy are required, such as, gastrointestinal endoscopy, and hematologic coagulation factor, hepatitis virus, and alpha-fetoprotein tests should be conducted to confirm the presence of varicose veins in the GI tract.

## Author contributions

**Conceptualization:** Jeong-Seok Choi.

**Data curation:** In Suh Park.

**Formal analysis:** Jeong-Seok Choi.

**Methodology:** In Suh Park.

**Supervision:** Jeong-Seok Choi.

**Validation:** Young-Mo Kim.

**Visualization:** In Suh Park.

**Writing – original draft:** Young-Mo Kim.

**Writing – review & editing:** Young-Mo Kim, Ji Won Kim, Jeong-Seok Choi.

Jeong-Seok Choi orcid: 0000-0001-9669-2141.
